# 4073 Parents of Faith Support School-Based Sex Education: A Louisiana Study

**DOI:** 10.1017/cts.2020.277

**Published:** 2020-07-29

**Authors:** Elise Walsh Boos, Caitlin Canfield, Lissane Brown, Kristie Bardell

**Affiliations:** 1 Albert Einstein College of Medicine; 2 Louisiana Public Health Institute; 3 Abt Associates

## Abstract

OBJECTIVES/GOALS: Louisiana state law does not require sex-education (SE) in public schools. Locally and nationally, religious identity and beliefs are often invoked to oppose access to sexual and reproductive healthcare and education. This study aimed to explore support for SE among Louisiana parents, focusing on how religiosity may influence parent support for SE. METHODS/STUDY POPULATION: Participants included 1,197 Louisiana parents and caregivers of children in grades K-12 who completed a web-based survey. Multivariate logistic regression analysis was used to determine associations between covariates and support for SE. RESULTS/ANTICIPATED RESULTS: Sixty-eight percent of parents reported that their overall approach to life is based on their religion or faith. Of those parents, 77% agreed that schools should be required to offer SE. In multivariate analysis, parents who reported that their whole approach to life is based on their religion on faith were 26% less likely to support required SE compared to parents whose whole approach to life was not based on their religion; however, this difference was not statistically significant (adjusted OR 0.74, 95% CI 0.44-1.24). Although support for required SE declined as religiosity increased, a strong majority of parents support requiring SE in Louisiana schools, regardless of religiosity. DISCUSSION/SIGNIFICANCE OF IMPACT: Contrary to opposition claims, strong support for SE exists among Louisiana parents and caregivers of faith. Parents and leaders of faith may be engaged as partners in advocacy for SE as well as other sexual and reproductive health issues.

